# *Candidatus* Rickettsia xinyangensis as Cause of Spotted Fever Group Rickettsiosis, Xinyang, China, 2015

**DOI:** 10.3201/eid2605.170294

**Published:** 2020-05

**Authors:** Hao Li, Xiao-Mei Li, Juan Du, Xiao-Ai Zhang, Ning Cui, Zhen-Dong Yang, Xiao-Jia Xue, Pan-He Zhang, Wu-Chun Cao, Wei Liu

**Affiliations:** State Key Laboratory of Pathogen and Biosecurity, Beijing Institute of Microbiology and Epidemiology, Beijing, China (H. Li, J. Du, X.-A. Zhang, P.-H. Zhang, W.-C. Cao, W. Liu);; ShanDong First Medical University and ShanDong Academy of Medical Sciences, Tai’an, China (X.-M. Li, X.-J. Xue);; People’s Liberation Army 154 Hospital, Xinyang, China (N. Cui, Z.-D. Yang);; Beijing Key Laboratory of Vector Borne and Natural Focus Infectious Diseases, Beijing (W. Liu)

**Keywords:** spotted fever group rickettsiae, rickettsiosis, human infection, *Haemaphysalis longicornis*, ticks, China, *Candidatus* Rickettsia xinyangensis, vector-borne infections, bacteria, *htr*A, *glt*A, *omp*A, *omp*B, *sca*4, phylogenetic analysis, field investigation, Rickettsia

## Abstract

In 2015, we evaluated 221 patients with undifferentiated fever and tick bite or animal exposure in Xinyang, China, for *Rickettsia* infection. Three with mild disease were infected with *Candidatus* R. xinyangensis, which clustered with *R. fournieri* and *R. vini* in phylogenetic analyses. Field investigations suggest *Haemaphysalis longicornis* ticks might be involved in transmission.

Spotted fever group (SFG) rickettsiae (SFGR) are obligate intracellular bacteria of the genus *Rickettsia* and family *Rickettsiaceae* and comprise >20 species identified as human pathogens (*1*). Most SFGR are transmitted by ticks (*1*), and flea-transmitted *R. felis* and mite-transmitted *R. akari* are recognized as members of the transitional group rickettsiae (*2*). In China, 4 different species and 1 new genotype of SFGR have been identified in association with human diseases (*3*,*4*).

Clinical symptoms of SFG rickettsioses are often simply fever and rash, although several other features, such as eschar and lymphadenopathy, are also commonly described (*1*). Diverse manifestations of diseases can make their clinical diagnoses rather difficult. Moreover, with the aid of molecular techniques, many new pathogenic SFGR are being discovered globally with increasing frequency. This increased discovery calls for researchers to intensify their efforts investigating patients with undifferentiated febrile illness. Here, we report a case series of 3 patients in China infected with the same novel SFG *Rickettsia*.

## The Study

During March–November 2015, we recruited 221 patients with undifferentiated febrile illness and history of tick bite or animal contact within the past month to a study conducted at the People’s Liberation Army 154 Hospital in Xinyang, Henan Province, China. We excluded patients with severe fever with thrombocytopenia syndrome virus infection (Appendix) and then tested for infection with SFGR.

We collected peripheral blood samples (using EDTA tubes) from patients at hospital admission and extracted DNA using the QIAmp DNA Blood Mini Kit (QIAGEN, https://www.qiagen.com). We concurrently performed nested PCRs specific for the conserved citrate synthase gene (*glt*A) and SFGR-restricted outer membrane protein A gene (*omp*A) (Appendix) (*4*). We then purified samples positive for amplicons and sequenced in both directions.

Three patients were found to be infected with a novel SFGR genotype with identical *glt*A and *omp*A gene sequences, which we designated *Rickettsia* sp. XY118. The *gltA* of XY118 (GenBank accession no. KU853023) had 99.6% (1,088/1,092) similarity with that of *R. vini* (accession no. KJ626330) and 99.6% (1,145/1,150) similarity with that of *R. heilongjiangesis* (accession no. CP002912) and *R. fournieri* (accession no. KF666471). The *ompA* gene sequence of XY118 (accession no. KU853021) was identical to those of undetermined *Rickettsia* species from ticks in China (accession no. AF169629) and Japan (accession no. AB516963) and rodents in South Korea (accession no. DQ402485). Moreover, the sequence of the *ompA* gene obtained in our patients had 96.1% (299/311) similarity with the corresponding gene in *R. vini* (accession no. KX159442) and 96.5% (335/347) similarity with that of *R. fournieri* (accession no. KF666477).

We collected serum samples from patients during the acute and convalescent phases of illness and tested for IgG against *R. rickettsii* by using an indirect immunofluorescence assay (Rickettsia IFA IgG; Focus Diagnostics, https://www.focusdx.com). Results showed that 2 patients had seroconverted and 1 had a 4-fold increased IgG titer (Appendix Table 2). In addition, we tested patients for acute infection with *Anaplasma phagocytophilum*, *Ehrlichia chaffeensis*, *Borrelia burgdorferi*, and *Babesia microti* by PCR and indirect immunofluorescence assay (*5*), and all blood samples were negative for both DNA of and specific IgG against these pathogens.

Two of 3 patients had reported history of tick bite, and 1 had reported animal contact (Table). All 3 patients had fever, asthenia, and anorexia. Two patients had eschar, 1 had lymphadenopathy, and none had rash. None of the 3 patients had any severe complications (i.e., hemorrhagic or neurologic signs or symptoms). Laboratory test results showed that 3 patients had leukopenia; 2 had thrombocytopenia; and 1 had elevated levels of hepatic aminotransferase, lactate dehydrogenase, and creatine kinase when admitted to the hospital (Appendix Figure 3). Clinical signs resolved and laboratory test findings were null (except for 1 patient with elevated hepatic aminotransferase levels) after 4–9 days’ hospitalization.

**Table:** Epidemiologic and clinical characteristics of 3 patients with *Candidatus* Rickettsia xinyangensis (XY118) infection, China, 2015*

Characteristics	Patient no.		
	1	2	3
Age, y	42	63	24
Sex	F	M	M
History of tick bite	Yes	No	Yes
Time from tick bite to disease onset, d	7	NA	7
Time from disease onset to hospital admission, d	3	4	5
No. days hospitalization	4	9	5
Signs and symptoms			
Fever	Yes	Yes	Yes
Highest temperature, °C	38.6	38.9	39.0
Dizziness	No	Yes	No
Asthenia	Yes	Yes	Yes
Myalgia	No	No	Yes
Eschar	Yes	No	Yes
Lymphadenopathy	No	No	Yes
Anorexia	Yes	Yes	Yes
Nausea	No	Yes	No
Cough	No	Yes	No
Rash	No	No	No

*NA, not applicable.

To identify local natural foci of SFGR, we performed a field investigation for infections among ticks captured around the 3 patients’ residences. We collected 232 host-seeking *Haemaphysalis longicornis* ticks and subjected each tick separately to DNA extraction with the DNeasy Blood & Tissue Kit (QIAGEN). *Rickettsia* sp. XY118 was detected in 2 (0.9%) ticks, and the nucleotide sequences of the *glt*A (GenBank accession no. KY617774) and *omp*A (accession no. KY617775) genes from these ticks were identical to those found in our patients. To further describe the genetic characteristics of this new genotype, we amplified the 16S rRNA gene (*rrs*; accession no. KY617772), 120-kDa genus common antigen gene (*omp*B; accession no. KY617776), PS120 protein–encoding gene (*sca*4; accession no. KY617777), and 17-kDa antigen gene (*htr*A; accession no. KY617773). The nucleotide sequence (1,320 bp) of *rrs* of XY118 had 99.7% (1,316/1,320) similarity with that of *R. japonica* (accession no. AP017602) and 99.6% (1,315/1,320) similarity with that of *R. heilongjiangesis* (accession no. CP002912; Appendix Figure 1). The nucleotide sequences of *htrA* (99.5%), *gltA* (99.6%), *ompA* (96.1%), and *ompB* (99.8%) from XY118 had the highest identity with the corresponding genes from *R. vini*. Compared with the partial *sca*4 sequence of *R. fournieri*, the corresponding sequence of XY118 contained 5 variable base pair sites and an 18-bp deletion (Appendix Figure 2). A phylogenetic tree that we constructed using the 2,546-bp nucleotide sequence of these 5 genes concatenated showed that *Rickettsia* sp. XY118, *R. fournieri*, and *R. vini* comprise a separate cluster that appears most closely related to *R. japonica* and *R. heilongjiangensis* (Figure). According to the gene sequence–based criteria for taxonomic classification of new *Rickettsia* isolates (*6*,*7*), a *Candidatus* status could be assigned to XY118, so we named this species *Candidatus* Rickettsia xinyangensis.

**Figure:**
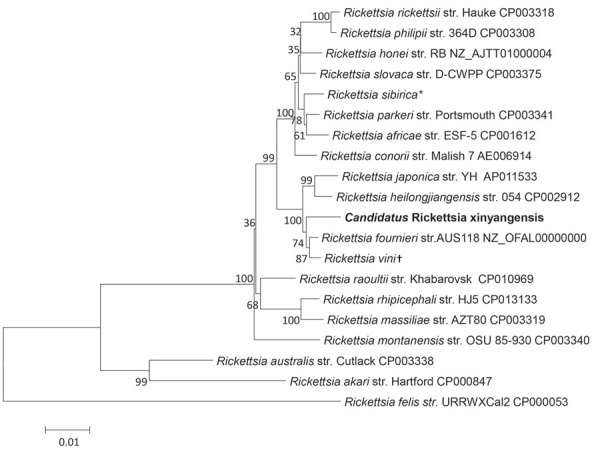
Phylogenetic analysis of concatenated nucleotide sequence of novel spotted fever group *Rickettsia*, *Candidatus* R. xinyangensis (bold), Xinyang, China, 2015. The partial nucleotide sequences of genes *htr*A (421 bp), *glt*A (1,092 bp), *omp*A (332 bp), *omp*B (456 bp), and *sca*4 (245 bp) were concatenated and compared via the maximum-likelihood method by using the best substitution model found (i.e., Tamura 3-parameter plus gamma) and MEGA version 5.0 (http://www.megasoftware.net). A bootstrap analysis of 1,000 replicates was applied to assess the reliability of the reconstructed phylogenies, and bootstrap values are indicated at branch nodes. Scale bar indicates the number of substitutions per nucleotide position. Str., strain. *Sequence includes the *htr*A gene from *R. sibirica* str. 246 (GenBank accession no. AABW01000001) and *glt*A (accession no. HM050296), *omp*A (accession no. HM050272), *omp*B (accession no. HM050273), and *sca*4 (accession no. HM050295) genes from str. RH05. †Sequence includes the *htr*A (accession no. KT187396), *glt*A (accession no. KT187394), and *omp*A (accession no. KT326194) genes from *R. vini* str. Breclav; *omp*B from str. 4GA09_32 (accession no. JF758826); and *sca*4 from str. IA-CR (accession no. KX159443).

## Conclusions

We found a novel SFG *Rickettsia* in human patients and ticks in China and propose the name *Candidatus* R. xinyangensis for this species. Our phylogenetic analyses involving comparisons with 5 different rickettsial genes showed that this newly identified SFG *Rickettsia* was most closely related to *R. fournieri*, a strain first isolated from *Argas lagenoplastis* ticks in Australia in 2013 (*8*) that has unknown pathogenicity in humans.

Our finding of *Candidatus* R. xinyangensis in 0.9% of *H. longicornis* ticks suggests a natural foci of this bacterium in Xinyang. However, extended field surveys and tick surveillance are required to understand the distribution of this agent and to identify specific tick vectors.

For *Candidatus* R. xinyangensis, a causal relationship between infection and clinical disease may be inferred by the serologic evidence, although only 3 patients infected with this pathogen have been reported. On the other hand, considering that isolates with identical (311-bp) SFGR-restricted *omp*A gene sequences have been detected in *H. yeni* and *H. longicornis* ticks in China (*9*,*10*), *H. longicornis* ticks in Japan (*11*), *H. bispinosa* ticks in Bangladesh (*12*), and *Apodemus agarius* rodents in Korea (*13*), *Candidatus* R. xinyangensis could be a tickborne infection of immense clinical relevance in humans.

In our study, patients with *Candidatus* R. xinyangensis infection had similar relatively mild febrile illnesses, as well as leukopenia and elevated hepatic enzyme levels, both of which are features of other SFG rickettsioses. In contrast, unlike patients with many other SFG rickettsioses, including Rocky Mountain spotted fever, our patients had eschars, not rashes (*1*,*14*). However, the patients described in this report were few in number and from a single hospital, and the true disease presentation of *Candidatus* R. xinyangensis infection might be more variable. Future investigations to further assess the disease spectrum of this pathogen and its contribution to clinical cases are needed.

Appendix More information on *Candidatus* Rickettsia xinyangensis as cause of spotted fever group rickettsiosis, Xinyang, China, 2015.
