# Supplementary material for: Candidatus Rickettsia xinyangensis as Cause of Spotted Fever Group Rickettsiosis, Xinyang, China, 2015
Source: Emerg Infect Dis. 2020 May;26(5):985–8. doi: 10.3201/eid2605.170294 (PMC7181907; doi:10.3201/eid2605.170294)
Supplement: Appendix — More information on Candidatus Rickettsia xinyangensis as cause of spotted fever group rickettsiosis, Xinyang, China, 2015. [file 17-0294-Techapp-s1.pdf]

Article DOI: https://doi.org/10.3201/eid2605.170294

Candidatus Rickettsia xinyangensis as Cause of Spotted Fever Group Rickettsiosis, China, 2015

Appendix

Severe Fever with Thrombocytopenia Syndrome Virus Detection by Real-Time PCR

Viral RNA was extracted from serum samples using QIAamp Viral RNA Mini Kit (Qiagen), according to the manufacturer's instructions. The real-time PCR was performed to detect severe fever with thrombocytopenia syndrome virus (SFTSV) RNA with the use of the One step Primer Script RT-PCR Kit (TaKaRa) as previously described (*1*).

Molecular Detection of Rickettsial Infection

DNA was extracted from blood specimens collected at admission with the use of the QIAamp Blood Mini Kit (Qiagen) according to manufacturer's instructions. Nested PCR assays targeting the *gltA* and *ompA* genes were concurrently performed to detect the presence of spotted fever group (SFG) rickettsial DNA. Nucleotide sequences of the primers were shown in Appendix Table 1.

To describe the genetic characteristics of this new genotype, the 16S rRNA gene (*rrs*), the 120-kDa genus-common antigen gene (*omp*B), the PS120-protein-encoding gene (*sca*4; gene D), and the 17-kDa antigen gene (*htr*A) were further amplified. Nucleotide sequences of the primers were shown in Appendix Table 1.

Appendix Results

Phylogenetic analysis based on concatenated datasets of *rrs* (1317 bp) partial nucleotide sequences was presented in Appendix Figure 1.

Serologic test results for 3 patients with *Candidatus* Rickettsia xinyangensis infection were shown in the Appendix Table 2.

Sequence comparison analysis of the 254 base-pair nucleotide sequence alignment of *sca*4 gene of *Rickettsia* was shown in Appendix Table 2.

References

1. Liu W, Lu QB, Cui N, Li H, Wang LY, Liu K, et al. Case-fatality ratio and effectiveness of ribavirin therapy among hospitalized patients in china who had severe fever with thrombocytopenia syndrome. Clin Infect Dis. 2013;57:1292–9. PubMed http://dx.doi.org/10.1093/cid/cit530

2. Mediannikov OY, Sidelnikov Y, Ivanov L, Mokretsova E, Fournier PE, Tarasevich I, et al. Acute tick-borne rickettsiosis caused by *Rickettsia heilongjiangensis* in Russian Far East. Emerg Infect Dis. 2004;10:810–7. PubMed http://dx.doi.org/10.3201/eid1005.030437

3. Regnery RL, Spruill CL, Plikaytis BD. Genotypic identification of rickettsiae and estimation of intraspecies sequence divergence for portions of two rickettsial genes. J Bacteriol. 1991;173:1576–89. PubMed http://dx.doi.org/10.1128/JB.173.5.1576-1589.1991

4. Jia N, Zheng YC, Jiang JF, Ma L, Cao WC. Human infection with *Candidatus* Rickettsia tarasevichiae. N Engl J Med. 2013;369:1178–80. PubMed http://dx.doi.org/10.1056/NEJMc1303004

5. Choi YJ, Jang WJ, Kim JH, Ryu JS, Lee SH, Park KH, et al. Spotted fever group and typhus group rickettsioses in humans, South Korea. Emerg Infect Dis. 2005;11:237–44. PubMed http://dx.doi.org/10.3201/eid1102.040603

6. Labruna MB, Whitworth T, Horta MC, Bouyer DH, McBride JW, Pinter A, et al. *Rickettsia* species infecting *Amblyomma cooperi* ticks from an area in the state of São Paulo, Brazil, where Brazilian spotted fever is endemic. J Clin Microbiol. 2004;42:90–8. PubMed http://dx.doi.org/10.1128/JCM.42.1.90-98.2004

Appendix Table 1. Nucleotide sequences of the primers used in the study

Target gene	Primer	Sequence (5’-3’)	Length of amplicon (bp)	Reference
*glt*A	CS1d	ATGACTAATGGCAATAATAA	1290	(*2*)
	CSEndr	CTTATACTCTCTATGTACA		
	RpCS877p	GGGGACCTGCTCACGGCGG	382	
	RpCS1258n	ATTGCAAAAAGTACAGTGAACA		
*omp*A	Rr190.70p	ATGGCGAATATTTCTCCAAAA	513	(*3*)
	Rr190.602n	AGTGCAGCATTCGCTCCCCCT		
	190.70-38s1	AAAACCGCTTTATTCACC	347	(*4*)
	190.602-384r1	GGCAACAAGTTACCTCCT		
rrs	RAF21	TTGCGTTAGCTCACCACCTT	1360	This study
	RAR1380	CACATGCAAGTCGAACGGAC		
	RAF886	ACCGCCTACGCACTCTTTAC	286	
	RAR1171	AAGCCGACGATCTGTAGCTG		
*omp*B	OF	GTAACCGGAAGTAATCGTTTCGTAA	503	(*5*)
	OR	GCTTTATAACCAGCTAAACCACC		
	SFG IF	GTTTAATACGTGCTGCTAACCAA	418	
	SFG/TG IR	GGTTTGGCCCATATACCATAAG		
*Sca*4	1072F	TCGGTTGAACCACCTCAGCATA	701	This study
	1782R	TGTGCCGGACTGAGAACTTGGA		
	1146F	GGCTTCACAAATGCCACAGTCG	389	
	1554R	TCTGCTGTTTTTGCTGCGGCTC		
*htr*A	17kd5	GCTTTACAAAATTCTAAAAACCATATA	548	(*6*)
	17kd3	TGTCTATCAATTCACAACTTGCC		
	17kd1	GCTCTTGCAACTTCTATGTT	434	
	17kd2	CATTGTTCGTCAGGTTGGCG		

Appendix Table 2. Serologic test results for 3 patients with *Candidatus* Rickettsia xinyangensis infection*

		Days after onset		IFA†	
Patient no.	Age/sex	AP	CP	AP	CP
Patient 1	42/F	4	21	<64	512
Patient 2	63/M	5	27	64	256
Patient 3	24/M	6	24	<64	128

*AP, acute phase; CP, convalescent phase; IFA, indirect immunofluorescence assay.

†Performed by detection of IgG antibodies against *R. rickettsii*.

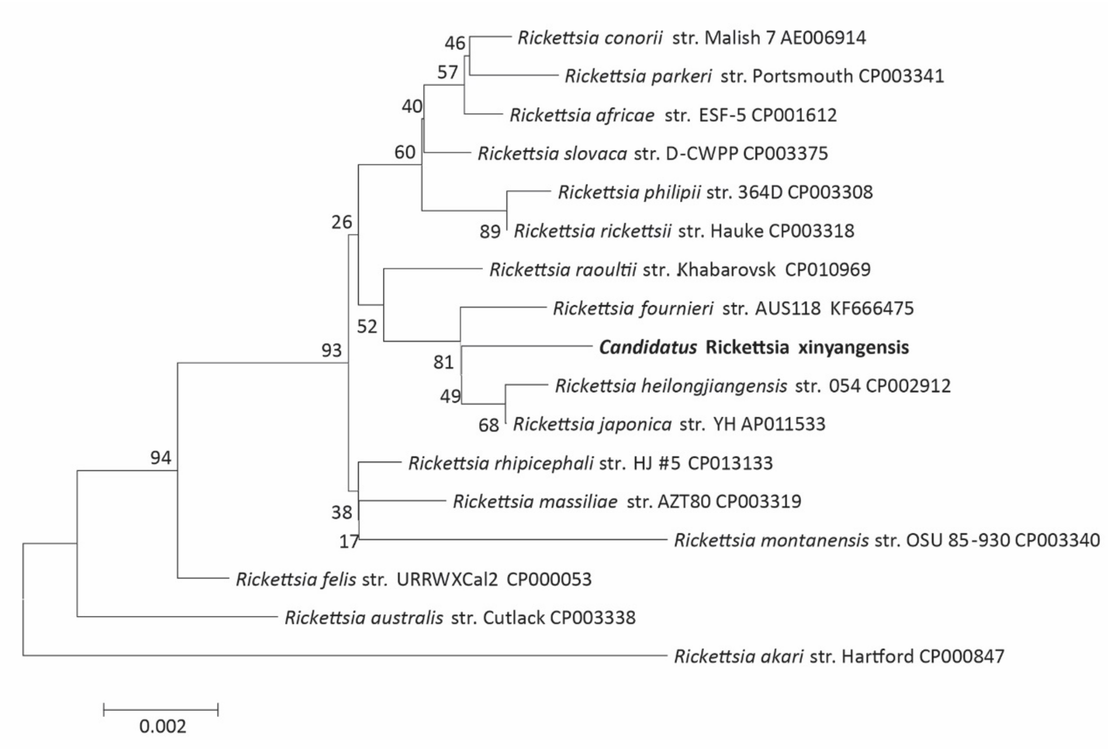

Appendix Figure 1. Phylogenetic analysis based on concatenated datasets of *rrs* (1317 bp) partial nucleotide sequences. The Maximum Likehood method with the best substitution model (Kimura 2-parameter + G) was conducted using MEGA version 5.0 (http://www.megasoftware.net). Bootstrap analysis of 1,000 replicates was applied to assess the reliability of the reconstructed phylogenies. Scale bars indicate the number of substitutions per nucleotide position. The strain of *Rickettsia* was specified. The bold indicates the novel species in the study (GenBank accession number KY617772).

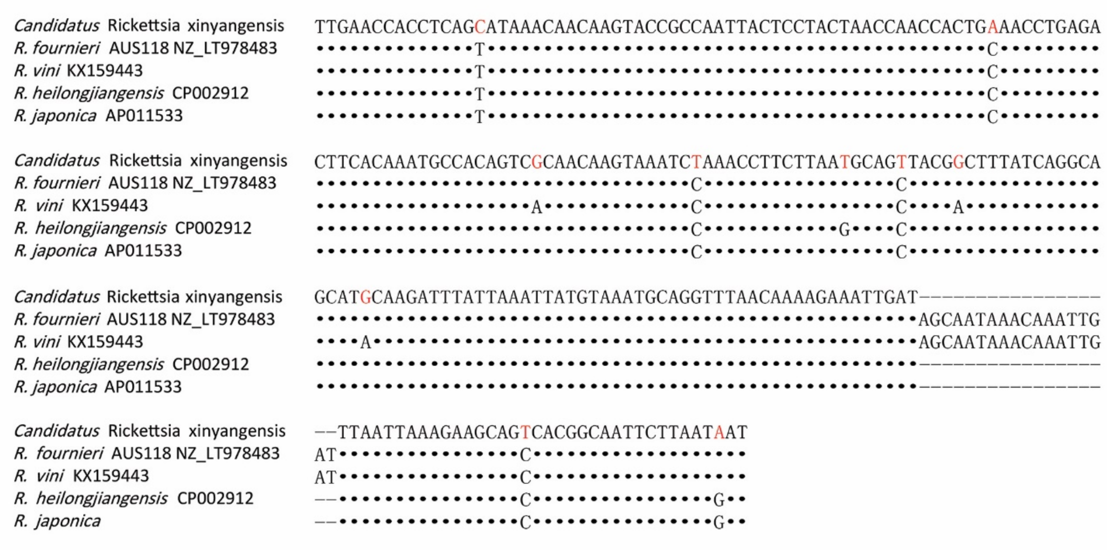

Appendix Figure 2. Sequence comparison analysis of the 254 base-pair nucleotide sequence alignment of *sca*4 gene of *Rickettsia*. The dot indicates the identical base, the red indicates different base, and the short line indicates base deletion.

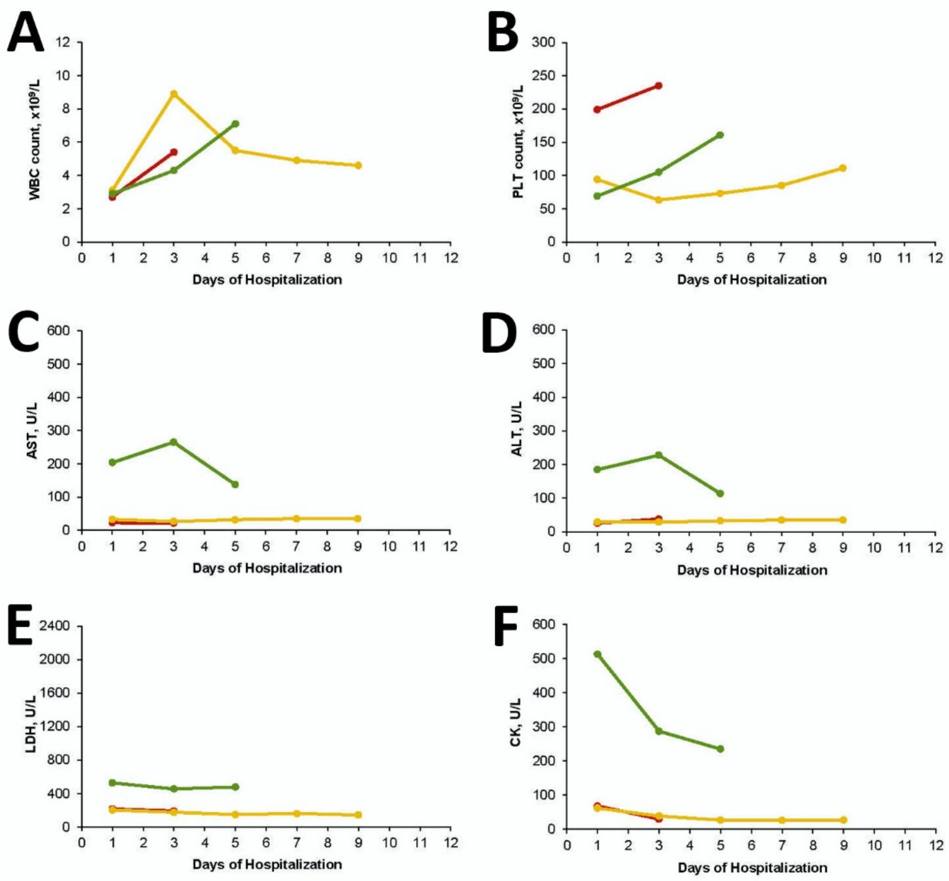

Appendix Figure 3. Dynamic changes of 6 laboratory parameters (with 2-day intervals) during hospitalization of 3 patients with *Candidatus* Rickettsia xinyangensis infection. Red, patient 1; yellow patient 2; green, patient 3. WBC reference range 4.0~10.5 x10^9 cells/L; PLT reference range 100~300 x10^9/L; AST reference range 0~40 U/L; ALT reference range 0~40 U/L; LDH reference range 109~245 U/L; CK reference range 25~200 U/L. ALT, alanine aminotransferase; AST, aspartate aminotransferase; CK, creatine kinase; LDH, lactate dehydrogenase; PLT, platelet; WBC, white blood cell.
